# Paradigm Shifts in Cardiac Pacing: Where Have We Been and What Lies Ahead?

**DOI:** 10.3390/jcm12082938

**Published:** 2023-04-18

**Authors:** Brennan A. Ballantyne, Derek S. Chew, Bert Vandenberk

**Affiliations:** 1 Department of Cardiac Sciences, Cumming School of Medicine, University of Calgary, Calgary, AB T2N 1N4, Canada; 2 Department of Cardiovascular Sciences, KU Leuven, 3000 Leuven, Belgium; 3 Department of Cardiology, University Hospitals Leuven, 3000 Leuven, Belgium

**Keywords:** pacing, electrophysiology, leadless, conduction system pacing, cardiac resynchronization therapy, left bundle branch pacing, His bundle pacing

## Abstract

The history of cardiac pacing dates back to the 1930s with externalized pacing and has evolved to incorporate transvenous, multi-lead, or even leadless devices. Annual implantation rates of cardiac implantable electronic devices have increased since the introduction of the implantable system, likely related to expanding indications, and increasing global life expectancy and aging demographics. Here, we summarize the relevant literature on cardiac pacing to demonstrate the enormous impact it has had within the field of cardiology. Further, we look forward to the future of cardiac pacing, including conduction system pacing and leadless pacing strategies.

## 1. Introduction

The history of cardiac implantable electronic devices (CIED) has been one of continued technological development and refinement to serve ever evolving clinical applications. After the initial description of syncope associated with a slowed pulse rate by Adams and Stokes, interest in device therapies for bradycardia began to flourish. The first pacing systems were developed in the 1930s and were externalized and operated by a hand crank to supply energy via a direct current generator. A needle was introduced directly into the right atrium after a transthoracic puncture was performed [1]. Through the 1940s and 1950s, transcutaneous pacing and transvenous pacing technologies were developed [2]. Eventually, an implantable system was developed to allow for the treatment of bradycardia in ambulatory patients [1]. Advances in material science, lead technology, sensor performance, programmable pacing/sensing algorithms, and implant procedures have since revolutionized the way clinicians treat and follow patients with bradyarrhythmias. Additionally, the advent of cardiac resynchronization therapy (CRT) has re-applied pacing therapies for the treatment of heart failure (HF).

## 2. Cardiac Pacing

### 2.1. General Description of Methods of Pacing

#### 2.1.1. Endocardial Pacing

A major leap forward in the history of cardiac pacing came with the introduction of transvenous pacing leads that could be implanted and fixed to endocardium. This allowed for more effective and reliable pacing and sensing compared to epicardial pacing. In most cases, endocardial transvenous leads connect to a pulse generator that is implanted into a surgical pocket created in the pectoral region. Various modes of venous access are available. 

#### 2.1.2. Epicardial Pacing

Original cardiac pacing devices utilized epicardial pacing techniques where an extravascular lead was implanted on the epicardium and connected to a pulse generator, originally implanted in the abdomen. Current epicardial pacing systems are used in patients with congenital heart disease, lack of venous access, history of recurrent device infections, or subjects who require CRT in whom a coronary sinus (CS) lead is not feasible. Current epicardial leads are implanted through various minimally invasive surgical techniques.

#### 2.1.3. Leadless Pacing

Technological innovations have led to the miniaturization of CIEDs and made leadless pacing possible. These percutaneous devices do not require surgical pocket formation or endovascular leads. Completely intracardiac systems can be deployed to the right ventricle for endocardial pacing using catheter-based implantation techniques. This technology is being rapidly adopted and holds significant promise for the future of cardiac pacing.

### 2.2. Introduction to Landmark Trials of Cardiac Pacing

The indications for cardiac pacing have expanded significantly since the initial reports describing pacing as a viable treatment option for the management of symptomatic bradycardia. Pivotal randomized controlled trials (RCT) of cardiac pacing for bradycardia are summarized in Table 1. The most common indications for pacing are high-degree atrio-ventricular block (AVB) and sinus node dysfunction (SND) [3]. There is no evidence that permanent pacemaker (PPM) therapy leads to a mortality benefit in patients with SND, but there can be clear improvements in symptoms and quality of life [4,5,6]. In contrast, patients with high-degree AVB have improved survival if treated with pacing therapy, when compared to those who do not undergo device implantation [3]. Regardless of pacing mode or indication for pacing, studies have uniformly demonstrated quality of life improvement after CIED implantation [7,8,9,10].

A major leap forward in our understanding of cardiac pacing came in the 1980s with the conceptualization of “physiological pacing” techniques to maintain AV synchrony and restore chronotropic competence with rate-responsive pacing technologies. A meta-analysis by Healey et al. revealed that atrial-based pacing is associated with 20% lower risk of incident atrial fibrillation (AF) and 19% lower risk of stroke compared to ventricular-based pacing [11]. No mortality benefits were observed. Other potential benefits may include improvements in exercise capacity, quality of life, and device diagnostic function. In patients with SND who are treated with dual-chamber pacing, optimization of the AV interval to maximize atrial pacing or native conduction and minimize ventricular pacing has been shown to reduce the risk of AF [12]. In patients with implantable cardioverter defibrillators (ICD), the addition of an atrial lead may be beneficial, particularly in patients with a coinciding indication for bradytherapy. It may also be easier to discriminate mechanisms of tachyarrhythmia in dual chamber systems compared to single chamber systems. However, in a meta-analysis comparing the differences in outcomes between single- and dual-chamber ICDs, an atrial lead was not associated with a reduced risk of inappropriate shocks [13].

### 2.3. Trends in Cardiac Device Implantation

Annual CIED implantation numbers have generally increased since the introduction of the implantable system, likely related to expanding indications, and increasing global life expectancy and aging demographics [14,15,16]. Currently, more than 80% of pacemakers are being implanted in patients over the age of 65 years [3]. The most recent estimates of worldwide annual implant rate are currently at approximately one million devices [14]. Most implants occur in high income countries, with rates of CIED implant over 1000 per million population [17]. According to the European Heart Rhythm Association (EHRA) 2017 white book, PPM implants have increased by 20% and ICD implants by 44% over a 10-year period in Europe [18]. Contemporary data on CIED implant incidence rates (per 100,000 people) over a 30-year study period (1988–2018) in Olmsted County, United States, reveal an overall CIED implant rate of 82.4 (95% CI 79.2–85.6) [16]. Implant incidence rates for PPMs were 62.9 (95% CI 60.0–65.7), for ICDs 14.0 (95% CI 12.6–15.3), and for CRTs 5.6 (95% CI 4.7–6.4) [16]. However, CIED utilization and implantation trends are far from uniform. An analysis of the EHRA 2017 white book results focusing on the impact of socioeconomic aspects of CIED applications revealed that European implant numbers varied significantly, with Germany implanting the most devices (196.53 implants per 100,000 inhabitants) and Kosovo the least (2.81 implants per 100,000 inhabitants) [19]. Higher implant numbers correlated with higher national gross domestic product (r = 0.456; *p* = 0.002) and higher health care expenditures (r = 0.586; *p* < 0.001) [19]. These utilization statistics are likely to progress in a more disparate way as new CIED technology is developed and adoption becomes even more challenging for less economically stable regions. Surely, this will be a future challenge for health care professionals and administrators. 

**Table 1:** Summary of Selected Landmark Cardiac Pacing Trials.

Trial	Year	Clinical Question	Intervention/Control	Population (N=)	Primary Outcome	Results	*p*-Value
CTOPP [20]	2000	What is the optimal pacing strategy for symptomatic bradycardia?	DDD/VVI	1474	Stroke, CV death	4.9 vs. 5.5% *	*p* = 0.33
MOST [21]	2002	What is the optimal pacing strategy for SND?	DDD/VVI	2010	All-cause mortality or non-fatal stroke	21.5 vs. 23% ^†^	*p* = 0.48
DAVID [22]	2002	What is the optimal pacing strategy for patients with standard indications for ICD without indications for pacing?	DDDR-ICD/VVI-ICD	506	Time to death or HFH	83.9 vs. 73.3 ^‡^	*p* < 0.03
UKPACE [23]	2005	What is the optimal pacing strategy for patients with high grade AVB?	DDD/VVI	2021	All-cause mortality	7.4 vs. 7.2% ^¶^	*p* = 0.56
DANPACE [24]	2011	What is the optimal pacing strategy for SND?	DDDR/AAIR	1415	All-cause mortality	27.3 vs. 29.6% ^§^	*p* = 0.53

SND = sinus node dysfunction; AVB = atrioventricular block; CV = cardiovascular; HFH = heart failure hospitalization; * AVB 60%, lower risk of AF (HR 0.82, *p* = 0.05) in DDD group, significantly more perioperative complications (*p* < 0.001) in DDD group; ^†^ Lower risk of AF (HR 0.79, *p* = 0.008) and lower HF scores (*p* < 0.001) in DDD group; ^‡^ Trial stopped early by DSMB, Trend towards higher HF hospitalization in DDDR group; ^¶^ No difference in AF, HF, stroke/TIA between groups; ^§^ Lower risk of AF (HR 0.73, *p* = 0.024) in DDD group, nearly double the pacemaker re-operation rate in AAIR group.

### 2.4. Cardiac Implantable Electronic Device Complication Rates

It is important to understand the complications associated with CIED implantation so that patients are well informed, but also to weigh the risks compared to the benefits of various CIED implantation strategies and select the best approach to individualize patient care and minimize the risks of harm. Table 2 provides an adapted summary of relevant CIED complications [25]. Compared to a single lead system, the addition of a right atrial pacing lead to a CIED system has been shown to increase the risk of procedural complications by 1.5–2 times [26,27]. This elevated risk is mediated by higher rates of lead dislodgement, pneumothorax and myocardial perforation [28].

CRT implantation is associated with higher peri-procedural risks compared to PPM or ICD implant. A systematic review and meta-analysis of 8 RCTs, including 5674 patients, revealed implantation success rates ranging from 88.3% to 98.4%, with an overall pooled success rate of 95.3% [29]. Peri-implant mortality was 0.4%. The pooled adverse event rate was 12.1%, compared to 5.3% in the ICD only group. This corresponded to a statistically higher rate of device-related complications compared to ICDs (OR 2.57; 95% CI 1.98–3.32). A total of 5.5% of patients experienced dislodgement or required lead repositioning for sub-optimal CS lead parameters. A recent study reporting complications associated with CS lead implantation demonstrates an improvement in complications rates over time: 10.7% between 2000 and 2004, and 3.2% between 2010 and 2014 [30].

Importantly, CIED systems are associated with a risk of infection ranging from 0.6–3.4% [25]. Patients who experience device infections are at increased risk of admission mortality (rate ratios 4.8–7.7; standardized rates 4.6–11.3%) and long-term mortality (rate ratios 1.6–2.1; standardized rates 26.5–35.1%) [31]. Additionally, length of stay and costs associated with device infection were substantially higher compared to those without infection [31,32]. An absorbable, antibiotic-eluting envelope has been developed that can be used at the time of CIED implantation in patients at risk of infection to substantially reduce infection rates. The WRAP-IT trial, a prospective randomized trial which evaluated the impact of this envelope, enrolled 6983 patients undergoing CIED pocket revision, generator replacement, or device upgrade [33]. Those patients who had prophylactic therapy with the envelope experienced a significant reduction in the primary endpoint of system extraction or revision for device infection compared to the control group (0.7% vs. 1.2%; *p* = 0.04). Envelope use has been shown to be cost effective when implant is guided by a CIED infection risk score [34].

Permanent transvenous leads pose a non-trivial lifelong risk of infective endocarditis (IE). A Danish nationwide registry analysis that included more than 40,000 patients who had undergone transvenous CIED system implants, with a total follow-up time of 168,343 person-years, found that the incidence of IE per 1000 patient-years ranged from 2.1 (95% CI 1.7–2.6) to 6.3 (95% CI 4.4–9.0) [35]. The lowest risk devices were single chamber pacemakers, and the highest risk were CRT defibrillators. The risk of death was significantly increased after CIED-related IE, with hazard ratios that ranged from 1.56 (95% CI 1.33–1.82) to 2.63 (95% CI 2.00–3.48), depending on device type [35].

There is significant heterogeneity in implantation techniques involving the venous access site and the mode of guiding vascular access. Each method has unique advantages and disadvantages that modify the risks of CIED implantation. The most common approaches to venous access are the subclavian vein puncture under fluoroscopic guidance and the cephalic vein cutdown. A review of almost 140,000 PPM implants from 2010 to 2014 revealed that the cephalic approach resulted in significantly fewer complications compared to the subclavian technique (2.49% vs. 3.64%; *p* = 0.0001) [36]. Although there were more pocket and vascular complications associated with cephalic cutdown, there were substantially lower rates of pneumothorax and lead failure. Fluoroscopically guided axillary venous access, compared to subclavian vein access, has been associated with similar rates of access success with significantly fewer perioperative complications and lead failures [37]. More recently, ultrasound-guided axillary venous punctures have been adopted by many CIED implanters. Success rates (80–99%) are comparable to fluoroscopically guided punctures and superior to the cephalic vein dissection techniques [38]. A prospective trial comparing ultrasound-guided approach versus traditional methods demonstrated lower time to access and total procedural times with no increase in complication rates [39].

## 3. New Developments in Cardiac Pacing

**Figure 1:**
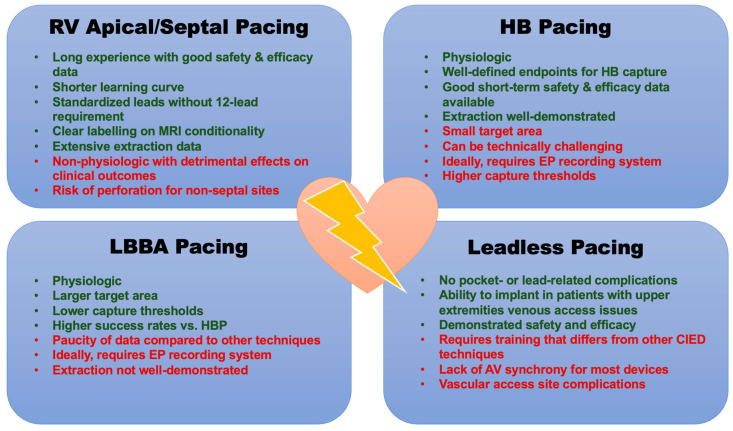
Various Methods of Cardiac Pacing. RV = right ventricle; HB = His bundle; LBBA = left bundle branch area; AV = atrioventricular.

We have come a long way since the advent of cardiac pacing and the introduction of transvenous systems. Thanks to new implantation techniques and technological developments, we are now observing several paradigms shifts in the established principles of cardiac pacing. Figure 1 provides a distilled summary of the various pacing strategies and their advantages and disadvantages. 

### 3.1. Cardiac Resynchronization Therapy

**Figure 2:**
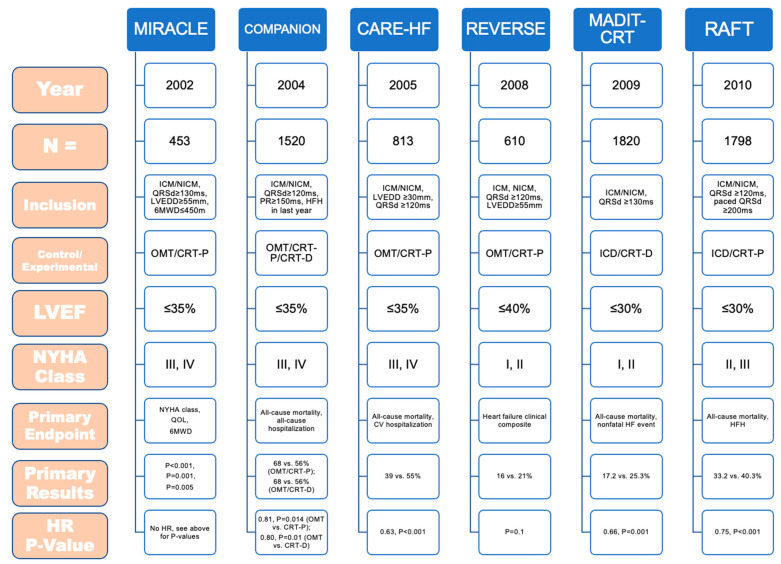
Results of Landmark Trials of Cardiac Resynchronization Therapy. ICM = ischemic cardiomyopathy; NICM = non-ischemic cardiomyopathy; QRSd = QRS duration; LVEDD = left ventricular end diastolic volume; 6MWD = 6-min walk distance; OMT = optimal medical therapy; CRT-D = cardiac resynchronization with defibrillation capabilities; CRT-P = cardiac resynchronization with pacing therapy only; NYHA = New York Heart Association; QOL = quality of life; HR = hazard ratio; HF = heart failure; HFH = heart failure hospitalization; CV = cardiovascular; MIRACLE [41]: CRT-P resulted in significant improvement in LVEF (*p* < 0.001); fewer HFH in CRT-P group (15 vs. 8%, *p* < 0.05); all patients in sinus rhythm; LBBB in almost 70% of patients; COMPANION [42]: CRT-P and -D both reduced the risk of the primary outcome by approximately 20%; no significant difference in mortality with CRT-P; addition of defibrillator significantly reduced risk of all-cause mortality by 36%; all patients in sinus rhythm; LBBB in almost 70% of patients; CARE-HF [43]: Patients with QRSd 120–149 must have 2 out of 3 for inclusion: 1. aortic pre-ejection delay >140 ms; 2. Interventricular mechanical delay >40 ms; 3. Delayed activation of PL LV wall; Significant mortality benefit (HR 0.64, *p* < 0.002); improved LVESVi, MR, LVEF, symptoms, QOL (*p* < 0.01 for all comparisons); proportion of LBBB not reported; all patients in sinus rhythm; REVERSE [50]: Heart failure clinical composite = All-cause mortality, HFH, crossover due to worsening HF, NYHA class, patient global assessment; patients with previous HFH were excluded; All patients in sinus rhythm; LBBB in more than 65% of patients; no difference in mortality; significant delay in time to first HFH in CRT group; CRT resulted in significant reverse LV remodeling. MADIT-CRT [44]: 41% reduction in HF events drove primary endpoint; no difference in ICM vs. NICM; most benefit for patients with QRSd ≥150 ms; decreased LV volumes, improvements in LVEF; no difference in mortality; all patients in sinus rhythm; LBBB in more than 70% of patients; RAFT [45]: Significantly more adverse events in the CRT group; sinus rhythm or permanent atrial fibrillation/flutter with controlled ventricular rates or planned total AVN ablation; LBBB in more than 70%. of patients.

A significant paradigm shift occurred with the adoption of pacing strategies in the form of CRT for patients with left ventricular (LV) dysfunction and symptomatic HF. Early data revealed that patients with systolic dysfunction who have electrical dyssynchrony (QRS duration ≥120 ms) have a 15% higher rate of mortality compared to those patients without dyssynchrony [40]. These findings were independent of age, severity of HF symptoms, and HF etiology. Electrical dyssynchrony as a marker of mechanical dyssynchrony has been linked to abnormal LV activation, suboptimal LV filling, increased myocardial work, reduced LV contractility, mitral regurgitation severity, maladaptive LV remodeling, and the development of an arrhythmic substrate. The positioning of a pacing lead within the CS to pre-activate areas of latest LV activation (usually the posterolateral LV wall) to achieve resynchronization, so called biventricular (BiV) pacing, has revolutionized HF therapy for carefully selected patients. Landmark CRT trials are highlighted in Figure 2. Initial RCTs enrolled highly symptomatic patients with LV dysfunction and QRS durations ≥120–130 ms, and compared CRT versus pharmacotherapy for LV dysfunction [41,42,43]. Patients in the CRT group experienced improvements in functional status, LV ejection fraction (LVEF), hospitalizations, and all-cause mortality compared to controls. Later trials that enrolled patients with less severe symptoms (NYHA class I–III) showed similar benefits [44,45]. The effects of CRT are strongest in selected patients with QRS prolongation in a left bundle branch block (LBBB) pattern [46].

A separate indication for CRT is the pace/ablate strategy, which describes implantation of a CIED for bradytherapy followed by total AV node ablation, in patients who have had difficulty with AF rate control [47]. The APAF-CRT trial was an open-label, blinded outcomes trial where 133 patients with highly symptomatic AF, narrow QRS, and at least 1 HF hospitalization in the previous year (regardless of LVEF) were randomized 1:1 to CRT plus ablation versus pharmacological rate control [48]. APAF-CRT consisted of two overlapping and consecutive phases, designated as the “morbidity” and “mortality” trials. In the morbidity trial, ablation plus CRT was associated with significantly fewer HF symptoms and HF hospitalizations [48]. The mortality trial was stopped prematurely for efficacy when all-cause mortality at a median 29 months of follow-up was significantly lower in the ablation plus CRT group compared to pharmacologically therapy (11% vs. 29%; HR 0.26, 95% CI 0.10–0.65; *p* = 0.004) [49]. The benefits of ablation plus CRT extended to all patients, regardless of LVEF.

Multiple studies have revealed that acute narrowing of QRS duration after CRT is associated with improved clinical outcomes, such as lower risk of all-cause mortality, urgent heart transplantation, and heart failure hospitalization [51,52]. Additionally, it has been demonstrated that LV pacing from sites of prolonged electrical delay is associated with improved response rates after CRT [53,54,55]. Measurements of intraventricular and interventricular delay can aid in the selection of an appropriate site for LV lead deployment during CRT implantation. A commonly used metric for this purpose is the QVL, which is measured from the time of QRS onset on the surface ECG compared to the first larger positive or negative peak on the LV electrogram [53].

Procedural failures related to LV lead placement during CRT implantation have been decreasing, yet finding an anatomically appropriate location for CS lead placement that is stable and affords adequate resynchronization remains challenging. In fact, this now accounts for over two-thirds of all procedural failures [56]. CS leads with active fixation technology have been developed to reduce these failure rates. The early generations of active fixation LV pacing leads were unipolar and relied on helices that could be expanded to “wedge” the lead into a CS branch (e.g., Attain StarFix, Medtronic, Minneapolis, MN). Observational data on early active fixation LV lead experience showed high procedural success (>95% success at non-apical locations), and low dislodgement rates (0.7%) at about 2 years [57]. Later generations of active fixation leads (e.g., Attain Stability, Medtronic, Minneapolis, MN) are quadripolar, and use small coaxial helices that can engage and fix within CS endothelium for enhanced stability. Again, observational data reveal very high procedural success (>98%) and no reported LV lead dislodgements [58]. However, observational studies have reported increased procedural complexity in extraction procedures for active fixation CS leads compared to their passive fixation counterparts [59]. Additionally, re-implantation in the same venous branch during the same procedure is often not feasible.

The Achilles Heel of CRT remains the high rate of non-responders, which is variably defined as failure to achieve HF symptom relief, improvements in LVEF, or LV dimensions. Rates of CRT non-response range from 30% to 50% of implants [60]. Through the ADVANCE CRT registry, Varma and colleagues found that CRT non-responders had higher rates of all-cause hospitalization (1.46 vs. 0.45 events per patient-year; *p* < 0.0001) and all-cause mortality (0.16 vs. 0.03 events per patient-year; *p* < 0.001) at 12 months post-implant [61]. While there are a number of potential approaches to combat CRT non-response, one that has gained attention recently is device algorithms for optimization of electrical resynchronization (Table 3). It is first important to understand that different combinations of programmed A-V and V-V delays can lead to numerous combinations of ventricular pacing wavefronts. There should be a personalized “optimal” setting that provides the best pacing timing to enhance CRT outcomes, which may be facilitated through CRT device algorithms that optimize resynchronization [62]. However, while demonstrating safety and non-inferiority compared to conventional BiV pacing or echocardiographic CRT optimization, multiple studies using available algorithms have not been able to demonstrate clear superiority in clinical outcomes [63,64,65,66,67]. The long-term follow-up data from the SMART-AV (NCT00677014), AdaptResponse (NCT02205359), and AutoAdapt (NCT04774523) algorithms are pending.

Multisite pacing (MSP) was proposed as a solution to CRT non-response. In experimental studies, MSP led to significant hemodynamic and electrophysiologic improvements [68]. MSP was initially achieved by implantation of separate LV leads in different CS branches with sufficient anatomic separation. The introduction of quadripolar CS pacing leads allowed for MSP via one CS lead implant. The MORE-CRT MPP trial enrolled 1921 patients with HF and LV dysfunction, who had a CS quadripolar lead implanted capable of MSP, and randomized to MSP compared to conventional BiV pacing [69]. MSP did not lead to a statistically significant difference in echocardiographic outcomes compared to conventional BiV-CRT, and the trial was ultimately terminated for futility. Despite these findings, quadripolar lead technology is now the preferred CS lead implant and is recommended as first-line for BiV pacing in CRT due to the ability to troubleshoot high pacing thresholds or phrenic stimulation [3].

Another emerging technique to improve CRT response rates involves targeted delivery of the LV lead. Taking into account that patients who have LV leads implanted in areas of scar have poor response to CRT [70], and that patients who have LV leads placed in areas of viable myocardium; especially with demonstrated late activation; produce high response rates [71], speckle-tracking 2D radial strain analysis by echocardiography has promise in the procedural planning of LV lead placement. Speckle-tracking software automatically analyzes the movement of stable patterns of natural acoustic markers, so-called speckles, and generates time-strain curves over the entire cardiac cycle. This allows for the identification of the latest area of LV activation, and the identification of non-viable myocardium (scar). The TARGET trial was an RCT with a total of 247 patients eligible for CRT, randomized to targeted LV lead positioning versus standard implantation [72]. The targeted group had a higher proportion of echocardiographic CRT responders at 6 months (70% vs. 55%; *p* = 0.031). Further, the targeted group had higher clinical response (83% vs. 65%; *p* = 0.003) and lower rates of the combined endpoint of mortality and HF hospitalization (*p* = 0.031). A longer-term follow-up study (median 39 months) revealed that targeted LV lead placement resulted in lower all-cause mortality (HR 1.8; *p* = 0.024) [73]. The STARTER trial had a similar design to TARGET and randomized 187 patients [74]. Targeted LV lead delivery resulted in lower risk of combined endpoint of first HF hospitalization or death (HR 0.48; 95% CI 0.28–0.82; *p* = 0.006) [74]. More recently, CMR-guided LV lead delivery has also gained interest. While larger, randomized trials are not yet available, a small observational study looked into the effects of CMR-guided LV lead placement [75]. Pre-procedural CMR studies generated 3D navigational models that allowed the LV lead to be directed to an area with the lowest scar burden, the most mechanical delay, and the farthest distance from the anticipated right ventricle (RV) lead location.

### 3.2. Conduction System Pacing

**Figure 3:**
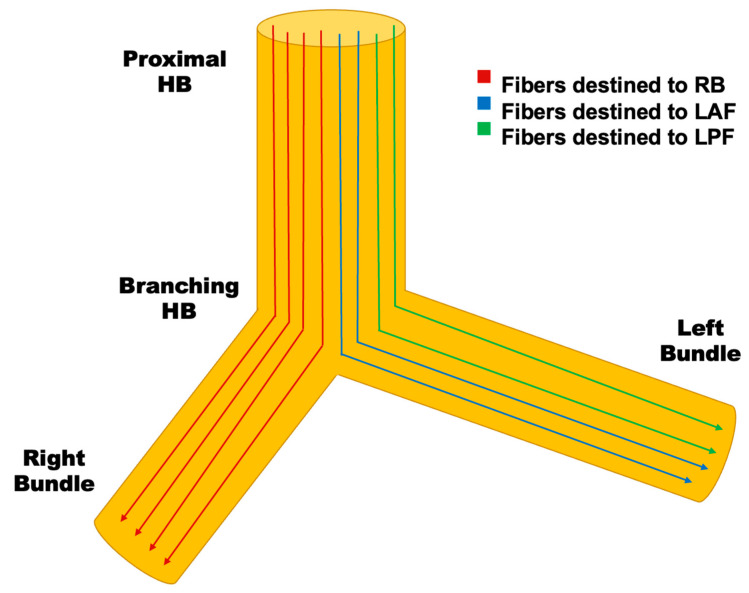
Schematic Demonstrating the Concept of Longitudinal Fascicular Dissociation. Histological studies have demonstrated that the His-Purkinje (HP) system is composed of multiple fibers with few transverse connections between fibers due to insulating collagen that runs along the longitudinal axis. A significant proportion of “infra-Hisian” block is in fact “intra-Hisian”. This explains how patients with conduction system disease (bundle branch block) can experience normalization of conduction when pacing at a more distal location within the HP system. HB = His bundle; RB = right bundle; LAF = left anterior fascicle; LPF = left posterior fascicle.

For almost 60 years, pacing from the RV apex has been the standard approach for long-term management of bradycardia given the demonstrated safety and efficacy of the procedure. However, RV apical pacing causes pre-excitation of the interventricular septum, and delayed activation of the lateral LV wall that results in electrical and mechanical dyssynchrony [76]. This translates clinically to pacing-induced cardiomyopathy in up to 20% of patients, and higher risk of HF hospitalizations over long-term follow-up [77]. Initial studies evaluating other pacing sites within the RV (non-selective septal pacing, RV outflow tract pacing) have shown limited success [78]. CRT has been the mainstay of device therapy for heart failure in patients with LV dysfunction for decades. Upgrading to CRT, however, may pose challenging access and an increased risk of complications [79]. Conduction system pacing (CSP), His bundle pacing (HBP), and left bundle branch pacing (LBBP), may overcome these challenges. CSP, based on the concept of longitudinal fascicular dissociation, describes the histological and electrophysiological properties of the His-Purkinje (HP) system as fibers that separate proximally in the His bundle (HB) and are predestined for more distal locations with very few transverse connections (Figure 3). This explains how pacing distal to the anticipated level of block can overcome conduction disturbances. Initial observations by Kaufman and Rothberger in 1919 of longitudinal fascicular dissociation within the His bundle were confirmed years later [80,81]. Scherlag et al. first verified the possibilities of selective capture of the HB [82,83]. Later, Deshmukh et al. demonstrated the clinical applicability of pacing the HB [84]. The results of early RCTs of CSP are summarized in Table 4.

#### 3.2.1. His Bundle Pacing

Initial attempts at implantation of pacing leads within the HB were conducted using standard leads with reshaped stylets, and mapping for the largest His deflection while recording intracardiac electrical signals. Although this resulted in capture of the His, initial studies revealed elevated pacing thresholds, acutely high rates of lead dislodgements, and procedural success rate under 70%, mostly related to failure to localize the His bundle [84]. Over time, specialized sheaths and leads have been developed for CSP applications. Early work uncovered the utility of HBP in patients with advanced AVB. One such study demonstrated that HBP was successful in 84% of unselected patients with AVB [85]. In 76% of cases, HP disease could be normalized completely, suggesting that HBP could be a strategy used routinely for bradycardia indications. This has since been supported by other observational trials [86,87].

**Table 4:** Randomized Controlled Trials Evaluating Conduction System Pacing Outcomes.

Study	His-SYNC [88]	His-Alternative [89]	LEVEL-AT [90]	LBBP-RESYNC [91]
Year of Publication	2019	2021	2022	2022
Type of CSP	HBP	HBP	HBP, LBBP	LBBP
Number of Patients	41	50	70	40
Age	64 ± 13	63.8 ± 9	65.7 ± 9	63.7 ± 11
Mean LVEF (%)	28	30 ± 6	27 ± 7	28.3 ± 5
Follow-up (mon)	6.2	6.0	6.0	6.0
LBBB (%)	85	100	60	100
Baseline QRS Duration (ms)	168 ± 18	165 ± 14	177 ± 21	174.6 ± 14
ICM (%)	65.0	20.0	31.4	0.0
Threshold (V)	1.7	2.4 ± 1.6	1.0 ± 0.4	0.82 ± 0.20
Pulse Width (ms)	1.0	1.0	0.6 ± 0.3	0.5
Procedure Time (min)	NR	137 ± 46	125 ± 35	129 ± 32
Complications (%)	NR	0	11.4% *	NR
Dislodgements (%)	0	0	1	0
Delta QRS duration (ms)	−28	−34	−53 ± 20	−43
Delta LVEF (%)	+9.1	+16 ± 7	+12.2 ± 9	+5.6
Delta LVESV (mL)	NR	−53	−37 ± 59	−25
Delta LVAT (ms)	NR	NR	−28 ± 26	79.74 ± 9.94 ^†^
Other Comments	No difference in CV hospitalization or death	---	No difference in mortality or HFH	BNP favoured LBBP

CSP = conduction system pacing; mon = months; ms = milliseconds; ICM = ischemic cardiomyopathy; V = volts; min = minutes; mL = milliliters; HBP = His bundle pacing; LBBP = left bundle branch pacing; NR = not reported; HFH = heart failure hospitalization; CV = cardiovascular; BNP = brain natriuretic peptide; * Requiring intervention; ^†^ Mean LVAT.

Initially, small observational studies demonstrated the low complication rates and effectiveness of HBP for resynchronization pacing in patients with HF [92,93,94,95]. Lustgarten et al. published the results of a prospective, randomized, cross-over study in which 29 patients with established indications for CRT were implanted with a system that included a CS lead for BiV pacing, and a HBP lead [96]. Patients had CRT for 6 months with BiV pacing, and were then changed to CSP for 6 months. Both BiV pacing and CSP had similar QRS narrowing, quality of life scores, functional outcomes, and echocardiographic changes, demonstrating real feasibility of HBP for CRT. In the largest observational multicenter cohort study, which included 844 patients who underwent HBP for bradycardia indications (41.2% AVB, 17.4% SND, and 39.7% bradycardia indications with AF), Zanon et al. reported long-term outcomes associated with HBP [97]. Only 1.7% of these patients had CRT indications. At a median follow-up time of 3 years, mean pacing threshold was 2V and sensed R-wave was 6.1 mV. Over 90% of patients were free from CIED complications at the end of follow-up. Currently, 5-year outcomes for patients with HBP are available. In a cohort study by Vijayaranan et al. HBP was attempted in 94 consecutive patients and was successful in 80% of cases [98]. Patients with HBP were less likely to experience death or HF hospitalization compared to RV pacing with >40% pacing burden (32% vs. 53%; *p* = 0.04). However, the need for lead revision (6.7% vs. 3%) and generator change (9% vs. 1%) were both higher in the HBP group. Pacing thresholds increased over time in both groups at 5 years compared to implant, but thresholds were significantly higher in the HBP group (1.62 V ± 1 vs. 0.84 V ± 0.4, respectively).

The efficacy of BiV-CRT is limited in patients with right bundle branch block (RBBB) and no pacing indication, and CSP has been proposed as an alternative CRT approach for this group of patients. In a prospective observational study, Sharma et al. report outcomes in patients with HF, LV dysfunction, and RBBB with QRS duration ≥120 ms who underwent HBP [99]. During a mean follow-up period of 15 ± 23 months, there was a significant reduction in QRS duration (127 ± 17 ms vs. 158 ± 24 ms; *p* = 0.0001), increase in LVEF (39 ± 13% vs. 31 ± 10%; *p* = 0.004), and improvement in NYHA functional class (2 ± 0.7 vs. 2.8 ± 0.6; *p* = 0.0001) compared to baseline.

A systematic review and meta-analysis of early HBP literature for patients who underwent device upgrade from conventional RV pacing to HBP for documented pacing-induced cardiomyopathy has been published by Zheng et al. [100]. This included 6 studies with a total of 144 patients. Over a mean follow-up period of approximately 18 months, patients who underwent device upgrade to an HBP system experienced improvements in LVEF (35 ± 8% vs. 48 ± 12%; *p* < 0.001), NYHA functional class (1.9 ± 0.8 vs. 2.7 ± 0.8; *p* < 0.001) compared to baseline. 

Several RCTs have compared ventricular resynchronization using HBP with CRT (Table 4). The His-SYNC trial assigned patients with guideline indications for CRT to HBP-CRT vs. BiV-CRT [88]. Patients who did not achieve adequate resynchronization with HBP, or who demonstrated elevated pacing thresholds (>5 V at 1 ms), were permitted to cross over. Additionally, when a LV lead could not be placed, crossover was also permitted. Crossover occurred in 48% of the HBP group, and 26% of the BiV pacing group. The HBP group demonstrated a statistically greater reduction in QRS duration, although the between-group difference was not significant. There was no difference in LVEF improvement, rates of CRT response, cardiovascular hospitalization, or death between groups. The thresholds after 6 months were higher in the HBP group. The His-Alternative trial randomized 50 patients with guideline indications for CRT with LBBB to HBP-CRT vs. BiV-CRT in a 1:1 fashion [89]. HBP-CRT pacing was successful in 72% of patients. At 6 months follow-up there was no significant difference in echocardiographic parameters between groups in the intentional-to-treat analysis. In the per-protocol analysis, the HBP group had higher LVEFs (48 ± 8% vs. 42 ± 8%; *p* = 0.014) and lower LV end systolic diameters (65 ± 23 mL vs. 83 ± 27 mL; *p* = 0.020). In meta-analyses which compared HBP vs. BiV pacing for CRT, HBP resulted in significantly better QRS duration narrowing (mean difference range −23.17–43.50 ms), LV activation time (LVAT), and LV dyssynchrony index, when reported [101,102]. No differences were noted between groups in echocardiographic parameters, functional test, quality of life, HF hospitalizations, or mortality.

#### 3.2.2. Left Bundle Branch Pacing

In 2017, the first case report demonstrating the clinical feasibility of LBBP was published by Huang et al. [103]. Initially HBP was attempted in a 72-year-old female with non-ischemic dilated cardiomyopathy, LV dysfunction, and LBBB, but due to high capture thresholds and failure to correct the LBBB the authors identified the location of the left bundle branch (LBB) with pace mapping. At low pacing outputs (0.5 V at 0.5 ms), correction of LBBB was achieved. They demonstrated selective LBB capture by varying the AV delays and stable outcomes at 1-year follow-up. The earliest observational study of LBBP in 11 patients with CRT indications was published in 2019 by Zhang et al. [104]. At a mean follow-up period of 6.7 months, QRS duration was significantly shortened (180.00 ± 15.86 ms to 129.09 ± 15.94 ms; *p* < 0.01). Additionally, NYHA class, cardiac biomarkers, and echocardiographic parameters had significantly improved compared to baseline. Other observational studies have corroborated these results, with high success rates of LBBP (85.0 to 97.8%) [105,106,107]. Therefore, LBBP has been investigated as a “rescue” strategy for CRT in patients who have had CS lead failures or CRT non-response. Vijayaraman et al. reported on 200 patients who underwent rescue CRT with LBBP [108]. This strategy resulted in significant QRS duration narrowing (170 ± 28 ms to 139 ± 25 ms; *p* < 0.001), LVEF improvements (29 ± 10% to 40 ± 12%; *p* < 0.001) after mean follow-up 12 ± 10.1 months. Risk of death or HF hospitalization was lower in those patients who underwent LBBP for CS lead failure compared to CRT non-responders (HR 0.357; 95% CI 0.168–0.756; *p* = 0.007). Resynchronization therapy using a LBBP strategy has been reported in a cohort of elderly patients as well. In a prospective, observational study, Grieco et al. enrolled patients aged 75 years and older with indications for CRT who underwent LBBP for resynchronization [109]. This cohort was compared to younger patients who also had CIEDs capable of LBBP implanted for similar indications. The outcomes were comparable between groups, with no significant difference in QRS narrowing, electrical parameters, LVEF improvement, or procedural complications over a follow-up period of 6 months.

Chen et al. reported on a prospective, multicenter, non-randomized, observational trial comparing the efficacy of LBBP against BiV pacing CRT with an adaptive BiV algorithm in 100 consecutive patients with HF and LBBB [110]. Success rates for CSP were higher, compared to BiV-CRT (98% vs. 91%). At 1 year, the change in LVEF was statistically different and in favour of the LBBP-CRT group. The pacing thresholds were also lower in the CSP group without a difference in procedure-related complications or clinical outcomes. The LBBP-RESYNC RCT included 40 patients with LBBB, LVEF ≤40%, and symptomatic HF due to NICM, who were randomized 1:1 to LBBP or BiV-CRT and followed for at least 6 months [91]. Procedural success rates were higher in the LBBP groups compared to BiV-CRT (90% vs. 80%). The QRS duration was significantly narrowed in both groups. LBBP resulted in greater LVEF improvement compared to BiV-CRT (mean difference 5.6%, 95%CI 0.3–10.9%; *p* = 0.039). Apart from LV end systolic volume and cardiac biomarker changes that favoured LBBP, there were no significant differences in other echocardiographic or clinical outcomes between groups. Similar findings were reported in the LEVEL-AT trial, a non-inferiority RCT including 70 patients with an indication for resynchronization therapy [90]. The first observational analysis to demonstrate a significant clinical benefit from CSP over BiV-CRT was recently published by Vijayaraman et al. [111]. A total of 477 patients who met indications for CRT at multiple centers were included. The primary outcome of death or HF hospitalization was significantly higher in the CRT group (28.3% vs. 38.4%; HR 1.52; 95% CI 1.082–2.087; *p* = 0.013). Wu et al. have reported the results of a non-randomized on-treatment comparison between LBBP versus HBP versus BiV-CRT (N = 137) [112]. Clinical and echocardiographic outcomes were similar between LBBP and HBP, and superior compared to BiV pacing.

In a recent multicenter international collaboration, Jastrzebski et al. reported outcomes of LBBP in a registry-based observational study [113]. This large (N = 2533) study included patients from 14 European centers who underwent LBBP for HF and bradyarrhythmia indications. The mean patient age was 73.9 years old, almost 60% of patients were female, and almost 30% had HF. Procedural success occurred in 92.4% of cases with a bradycardia indication, and 82.2% of cases with a HF indication. At a mean follow-up of 6.4 months, capture thresholds (0.77 V) and sensing (10.6 mV) were stable. Complications occurred in 11.7% of cases, with complications specific to the transseptal approach for lead placement in 8.3% of cases [113]. 

#### 3.2.3. Conduction System Pacing Implantation Techniques

##### General

**Figure 4:**
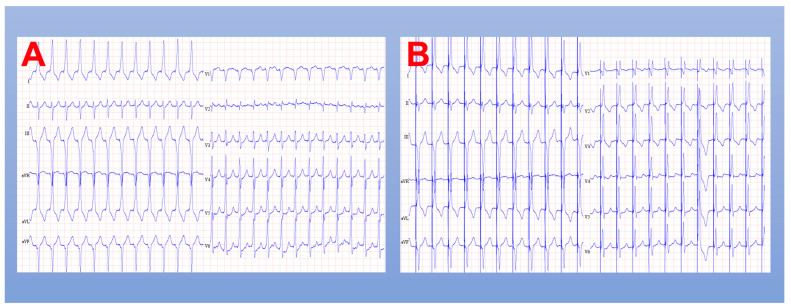
ECG results after conduction system pacing. (**A**) Left bundle branch pacing (bipolar); (**B**) left bundle branch pacing (unipolar).

In both HBP and LBBP procedures, continuous intracardiac electrogram and 12-lead ECG recording should be utilized, ideally with an EP recording system. After venous access is obtained, a specialized sheath shaped to direct the lead to the HB or LBB area is advanced over a guidewire through the tricuspid valve, the RV, and to the RV outflow tract. Once the guidewire is removed, the sheath can be withdrawn to the approximate location of interest and a pacing lead is advanced through the sheath so just the helix is exposed. This allows for unipolar mapping of local intracardiac electrograms to more precisely find the appropriate location to begin lead fixation. Once this location is determined, an active fixation lead is deployed, and lead parameters are checked in both unipolar and bipolar modes (Figure 4).

##### His Bundle Pacing

In HBP, both atrial and ventricular components of the membranous septum can be interrogated for a His signal during unipolar sensing. Several permutations to the implantation technique have been described to guide localization to the area of the His. The use of contrast injection to visualize the tricuspid valve annulus has been shown to reduce fluoroscopy times [114]. Fluoro-less or nearly fluoro-less procedures, by using exclusively intracardiac electrograms to guide lead implantation, have also been reported [115,116]. Once a HB potential is visualized, unipolar pacing is performed to assess capture responses and confirm optimal location for lead implantation. A detailed review of the pacing responses during HBP is beyond the scope of this review. In general terms, the following capture responses can be observed based on the QRS morphology while pacing in the region of the HB:

1. Selective response: capture of the HB alone, with ventricular capture exclusively through the conduction system;
2. Non-selective response: activation of HB and local myocardium. Ventricular capture results from fusion of both wavefronts.

Although selective His capture is ideal, there have been studies demonstrating preservation of electromechanical synchrony with non-selective HB capture [117,118].

##### Left Bundle Branch Pacing

In selected cases, a pre-implantation echocardiogram may be helpful to assess the thickness of the interventricular septum for procedural planning. Similar to the HBP approach, unipolar sensing is utilized, and a specialized introducer sheath can help guide leads to the area of interest. The sheath is positioned distal to the location of the HB in the right anterior oblique view and faces 1 to 2 o’clock position. Fluoro-less approaches to LBBP have been described [119]. Pace mapping with unipolar pacing helps confirm a septal pacing location, including looking for:

1. V1—QS complex with notch in the descending limb near the nadir (“W” complex);
2. Tall R-waves in leads II, III (ideally, II > III);
3. Discordant QRS in leads aVR (negative) and aVL (positive).

Lead deployment can be performed, guided by two outcomes:

1. Gradual deployment while monitoring the paced QRS morphology and impedance;
  - Gradual increase in R’ wave in V1 with progression of R wave to the terminal component of the QRS (Qr pattern or rSr’ pattern);
  - Gradual increase in impedance before drop of 100–200 Ohms prior to LV subendocardium;
  - Myocardium current of injury;
2. Rapid deployment with monitoring of PVC morphology;
  - PVC morphology changes from wide QRS to narrowed QRS with RBBB morphology (duration < 130 ms).

Through this process, a LBB potential may be visualized, which indicates an ideal spot. A drop in paced impedance of more than 200 Ohms, unipolar impedance of <400 Ohms, or poor R-wave sensing with diminished current of injury suggests perforation through to the LV endocardium. Programmed extra stimulus testing can be performed to differentiate LBB capture from septal myocardial capture based on differences in refractory periods. Jastrzebski et al. have described an approach that uses an 8-beat drive train at 600 ms with an extra stimulus with coupling interval at 450 ms [120]. The extra stimulus is decreased by 10 ms intervals and the response can be categorized as myocardial (non-selective) or selective LBB capture, based on criteria presented above. The left ventricular activation time (LVAT) is defined as the duration between stimulus to peak R wave in leftward precordial leads (V4-V6), and is a measure of the rapidity of activation of the lateral LV wall. The LVAT should abruptly shorten during LBB capture and is a good marker of an effective location. During lead deployment, contrast angiography of the septum (“septogram”) can also be performed to determine the appropriate location for lead implantation, or to assess the depth of lead implantation post-lead fixation. Commonly used leads have defined measurements from the end of the active fixation helix to more proximal radiopaque markers to allow estimation of septal depth. Specific endpoints that can be used to suggest a successful LBBP location include:

1. RBBB pattern during pacing;
2. Presence of LBB potential during pacing (visualized in less than 50% of cases);
3. Short and constant LVAT high- (5 V) and low- (1 V) pacing outputs;
4. Demonstration of selective and non-selective LBB pacing;
5. Evidence for direct LBB capture.

### 3.3. Leadless Pacing

**Figure 5:**
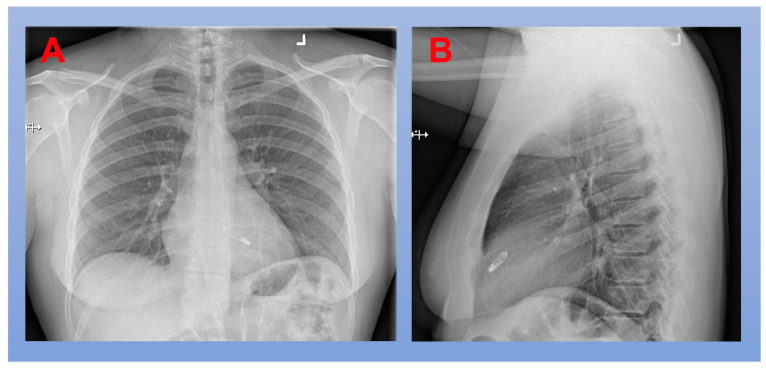
Chest X-Ray After Implantation of a Leadless Pacemaker. (**A**) PA projection; (**B**) lateral projection.

As previously outlined, the complications related to CIED systems that utilize transvenous leads are not trivial. Leadless pacing was developed to eliminate both pocket- and lead-related device complications (Figure 5). A percutaneous, catheter-based introducer system employs venous access (generally through a femoral vein) to allow for delivery of a leadless CIED which can be deployed at various locations in the RV, including septal (most common), apical, and outflow tract. Leadless pacemakers (LP) have been developed that use passive fixation (Micra™, Micra AV™; Medtronic, Minneapolis, MN, USA) and active fixation (NanoStim™, Aveir™; Abbott, Abbott Park, IL, USA) mechanisms. The NanoStim LP was withdrawn from the market between 2016 to 2017 after battery malfunctions that caused problems with telemetry and pacing, as well as reported detachments of the docking button. Each LP had its pivotal trial (LEADLESS II, Abbott NanoStim; Reynolds et al., Medtronic Micra; LEADLESS-II-phase 2, Abbott Aveir) reporting implant success rates above 95% and successfully meeting the predefined primary efficacy and safety endpoints. For a detailed summary of landmark LP trials, refer to Table 5.

Despite these very promising efficacy and outcomes studies, one significant limitation of LP was the lack of AV synchrony. The first LP trials strictly enrolled patients with an indication for single-chamber pacing. In fact, most trials had a very high representation of patients with AF-related bradycardia and pacing indications. The Micra AV LP takes advantage of a three-axis accelerometer that can sense atrial contractions and time a ventricular pacing event to maintain AV synchrony. A device algorithm has been developed that disregards motion related to AV valve and semilunar valve closures, ventricular diastole, and passive ventricular filling, but can sense mechanical events related to atrial contraction. This initiates a programmed AV delay, hence providing VDD pacing in essence. The initial feasibility study (MARVEL 1) of the AV algorithm was published in 2018 and demonstrated 87.0% (95% CI 81.8–90.9%) AV synchrony at rest [125]. Examination of Holter data from this patient population confirmed that the algorithm did not lead to pauses, or tachycardia related to oversensing. Refinements to the algorithm following the feasibility analysis included automated programming features and a mode switch feature. The MARVEL 2 trial was the next feasibility analysis using the updated device algorithm [126]. In this prospective multicenter trial, 75 patients were enrolled, 40 of which had sinus rhythm with complete AV block and were included in the efficacy analysis. The mean percentage of AV synchrony was 89.2% (95% CI 84.8–92.5%) in this group [126]. In the larger and more recent AccelAV study, 152 patients were implanted with the Micra AV device, and AV synchrony at rest was 85.4% (95% CI 81.1–88.9%) during VDD pacing [127]. The reasons for low AV synchrony (<70%) included: high resting heart rates, variable heart rates, low-amplitude sensed signal, and suboptimal programming. In a real-world analysis of 20 patients, including 816 h of Holter ECG data and treadmill exercise testing, those who were predominantly paced with sinus rates of 50–80 bpm had a median AV synchrony of 91% (IQR 34–100%) [128]. However, the median AV synchrony was significantly lower when patients had sinus rates over 80 bpm (33%; *p* < 0.001); nevertheless, device optimization through serial clinic follow-ups was able to improve AV synchrony. An analysis of patients enrolled in the MARVEL 2 trial found that the strongest predictors of high AV synchrony (>90% correct atrial-triggered ventricular pacing) were an E/A ratio of <0.94 on echocardiogram, and low sinus rate variability at rest [129]. Currently, the Aveir DR i2i study is enrolling patients (NCT05252702) to evaluate the safety and efficacy of implantation of the Aveir dual-chamber leadless pacing system in patients with indications for both atrial and ventricular pacing.

Over time, LP have demonstrated significant safety advantages over transvenous systems. In a systematic review and meta-analysis that pooled the results of 36 observational trials, over 98% of patients had adequate pacing thresholds at 1 year [130]. The pooled incidence of complications at 1 year was 1.77% (95% CI 0.76–3.07%) and LP was associated with 51% lower odds of complications compared to transvenous CIED systems. A retrospective cohort study of 155 patients who underwent Micra LP implantation found 15 patients who developed bacteremia at a median of 226 days post-implant [131]. No pacemaker endocarditis was observed in these patients. In a sub-analysis of the Micra post-approval registry, 105 patients underwent LP implantation within 30 days of extraction of an infected transvenous CIED system [132]. No patients developed LP infection during a mean follow-up duration of 8.5 ± 7.1 months. Similar data have been observed from other LP devices [122]. In fact, confirmed infections of LPs are so rare, the literature is limited to case reports and case series data [133]. There are even data to support the safety of LP implantation with concomitant transvenous lead extraction during active CIED infection [134,135]. Breeman et al. have described the longest follow-up study of patients who underwent LP implantation after CIED extraction for device infection [136]. They report outcomes of 29 patients after a median 2.5 years of follow-up after LP implant, 30% of which were implanted prior to or during extraction of the infected CIED system. No re-infections occurred during the follow-up period [136]. It has been posited that the reason for the exceedingly low risk of LP infection is related to several important differences in LP engineering compared to transvenous devices [137]. These include:

1. Absence of subcutaneous pocket;
2. Substantially reduced device surface area;
3. Minimal physical handling of device pre-implant;
4. Extensive encapsulation of device after implant;
5. Turbulent hemodynamic environment with high-velocity blood flow;
6. Parylene-coated titanium material that may reduce bacterial adherence.

## 4. Future Directions

### 4.1. Expanding Applications for Conduction System Pacing

The feasibility and efficacy of CSP for patients undergoing AVN ablation as a component of a pace/ablate strategy for AF management has been demonstrated in several small, observational studies [138,139,140,141]. Recently, the feasibility of CSP combined with total AV node ablation has been described in an observational study which included patients with refractory AF who were referred for pace/ablate strategy from 2015 through 2020 (single center) [142]. Conventional RV pacing versus CSP was performed at operator discretion. A total of 223 patients (CSP 110) underwent AVN ablation after CIED implantation. The mean LVEF was 43% (±15%). Over a mean follow-up period of 27 months, QRS durations were shorter in the CSP group, and LVEF were higher in the CSP group. The combined endpoint of time to death or heart failure hospitalization was reduced in CSP compared to the conventional pacing group (48% vs. 62%; *p* < 0.01). A retrospective study comparing outcomes in patients with AF and HF, who underwent CSP (n = 37) versus BiV-CRT (n = 13) and subsequent AV node ablation, revealed that NYHA class improved in the CSP group irrespective of CSP approach (HBP: *p* < 0.001; LBBP: *p* = 0.008), but not in the BiV group (*p* = 0.096) [143]. Additionally, LVEF improved in the CSP group (HBP: *p* < 0.001; LBBP: *p* = 0.041), and not in the BiV group (*p* = 0.916). The CONDUCT-AF trial (NCT05467163) will be a multicenter RCT; it plans to enroll patients with AF and HF with narrow QRS durations who are appropriate for pace/ablate strategy, and aims to randomize to CRT with BiV pacing compared to CSP.

CSP holds significant promise, but large comparator studies are required to confirm the safety and efficacy that has been suggested by smaller exploratory studies. Two future trials will evaluate the application of CSP for patients at risk of pacing-induced cardiomyopathy or with HF, both comparing CSP strategies to established therapies in larger RCT formats. In the LEFT-HF trial (NCT05015660), investigators plan to recruit at least 100 patients with normal to mild LV dysfunction and high grade AVB, and randomize to LBBP versus RV apical pacing. Primary outcome measures will include LV dimensions on echocardiography and implant success. In the “Left Versus Left” trial (NCT05650658), investigators aim to recruit more than 2000 patients with HF and either a wide QRS or an anticipated pacing burden of greater than 40%. Patients will be randomized by receiving CSP (HPB or LBBP) versus BiV pacing for resynchronization. The primary outcome will be a combination of all-cause mortality and hospitalization for HF.

### 4.2. Extraction of Conduction System Pacing Leads

The first reported case of lead extraction of a LBBP system was published by Vijayaraman et al. in 2020 [144]. They describe a patient who required CIED system extraction for gram positive bacteremia. The lumen-less pacing lead was successfully extracted about 1 year after implant with gentle traction only, and no complications. Since this time, other case reports have been published which demonstrate feasibility and safety of LBBP lead extraction, albeit with all leads less than 2 years old and only manual traction required for extraction [145,146]. There has been considerably more experience with extraction of HBP leads. One study which included 30 patients with HBP leads more than 6 months old (mean age 25 ± 18 months) demonstrated 100% extraction success with durations ≤12 months, and 95% success with durations >12 months. Extraction tools were required in only four patients [147]. The use of stylet-driven leads for CSP has been compared to lumen-less leads and has been shown to be feasible [148]. However, case reports exist documenting early sudden distal conductor fractures when using stylet-driven leads [149]. This may have implications for lead extraction. One case report documented a femoral approach with double snare technique to extract such a lead where the conventional stylet approach was not possible due to conductor fracture [150]. Overall, experience with extraction of CSP leads is limited and we require more data as the volume of implanted CIED systems utilizing CSP increases worldwide.

### 4.3. Leadless Pacing Combined with Subcutaneous Implantable Cardioverter Defibrillators

Subcutaneous implantable cardioverter defibrillators (S-ICD) have been developed to avoid the complications associated with transvenous lead implantation, while maintaining the ability to treat life-threatening ventricular tachyarrhythmias [151,152]. While S-ICDs have certain advantages over conventional transvenous ICD systems, they are inappropriate for patients with slower, monomorphic VT requiring anti-tachycardia pacing, or patients who also require bradycardia therapy due to the absence of transvenous leads. However, the combination of S-ICD and LP implantation may be an effective way to deliver both tachycardia and bradycardia therapy, while avoiding transvenous lead implantation. In a recently published pre-clinical study, Breeman at al. describe implantation of S-ICD and LP systems in 38 canine subjects with 100% procedural success and excellent long-term pacing/sensing parameters [153]. This looks to be a promising new frontier for the treatment of ventricular arrhythmia.

### 4.4. Optimizing Cardiac Resynchronization

In addition to resynchronization through QRS narrowing by HBP, the utility of this mode of pacing has been investigated as an adjunction to traditional CRT with left ventricular endocardial pacing with a CS lead. This so-called “His-Optimized CRT” (HOT-CRT) has been studied for a subset of patients who had CRT indications and had failed to completely correct HP conduction abnormalities with HBP alone, or who were clinical or echocardiographic non-responders to HB pacing. The feasibility study by Vijayaraman et al. enrolled 27 patients to HOT-CRT pacing [154]. In 93% of cases, HOT-CRT was successful, resulting in significant improvements in QRS duration, echocardiographic parameters, and NYHA class. This response was observed in three out of four of the CRT non-responders. These results have since been replicated by other small observational studies [155].

Similar to HOT-CRT, achieving resynchronization through pacing the LBB has led to investigation into the feasibility of “Left Bundle Branch-Optimized Cardiac Resynchronization Therapy” (LOT-CRT). Initial feasibility data were published by Dr. Vijayaraman in 2021 [156]. The largest, prospective observational study of LOT-CRT was a multicenter study that included 112 patients with CRT indications who had either failed BiV-CRT or were selected for de novo LOT-CRT at the operators’ discretion [157]. LOT-CRT was successful in 81% of patients and resulted in significantly more QRS duration narrowing compared to BiV- or LBBP-CRT alone. At follow-up more than 3 months post-implant, there was an improvement in echocardiographic parameters and HF biomarkers compared to baseline.

### 4.5. The WiSE-CRT System

The WiSE-CRT (EBR Systems, Sunnyvale, CA, USA) system is a promising application of leadless pacing that attempts to deliver endocardial LV pacing via a very small (9.1 × 2.6 × 3.6 mm) anchoring electrode implanted onto the LV endocardium to achieve CRT. In addition to the LV electrode, the system requires a small transmitter implanted at a pre-determined intercostal space, and a generator implanted at the left mid-axillary line [158]. The original design required implantation of the LV electrode at the lateral wall for CRT, but implantation onto the LV septum to capture the LBB to achieve biventricular synchrony has now been described [158]. It has been successfully implemented in animal models and human patients, with significantly reduced QRS duration, durability at short term follow-up, and symptomatic improvements [158,159]. While this still requires an RV transvenous lead, this is a promising paradigm shift that may allow for leadless resynchronization therapy in the future, especially when paired with developing S-ICD technology.

### 4.6. Future Economic Perspectives

It will be important to bear in mind the economic challenges that accompany ongoing technological developments in the field of CIED implantation. In general, as technologies mature, there is a steady decrease in the price of the associated goods [160]. This trend has been reported for the costs of ICDs. Na et al. conducted a single-center (U.S.) review of the costs associated with a hospitalization for ICD implantation that included encounters from 2015–2019 [161]. Cost analysis revealed a modest (− USD 1.82/day; *p* < 0.001) decrease in the supply costs of ICD implantation over this period. However, there was no significant decrease in the overall costs associated with these encounters, meaning that other costs were rising to offset improving supply costs [161]. Technological developments are progressing rapidly, and costs may not have time to drop with increasing adoption of new products. As a result, we may witness a rise in overall costs associated with the management of patients who require CIEDs. Additionally, the indications for CIED implantation continue to expand and evolve, adding additional economic pressures to health care systems. A particularly illustrative example of this is observed in the increased CIED implantation rates associated with transcatheter aortic valve implantation (TAVI). Damage to the conduction system during TAVI procedures results in the need for PPM implantation in about 10–14% of cases (compared to under 3% of surgical aortic valve interventions) [162,163]. As CIED indications continue to evolve, it will become increasingly important for practitioners and administrators to responsibly control costs and continue to analyze the cost-effectiveness of various CIED management strategies.

## 5. Conclusions

Cardiac pacing has a long and storied past with several revolutions and paradigm shifts. The introduction of new technologies, procedural techniques, and increasing demand for new clinical applications for pacing therapy has pushed the field of cardiac pacing forward. From this vantage point, the future looks bright.

## Figures and Tables

**Table 2:** Complications Associated with Implantation of Cardiac Implantable Electronic Devices.

Complication	Incidence
Mortality (procedure-related)	<0.1%
Pneumothorax	0.4–2.8%
Perforation	0.1–1.5%
Cardiac tamponade	0.5–1.5%
Pocket hematoma	0.2–16.0%
Infection	0.6–3.4%
Lead dislodgement	1.2–3.3%
Other	<0.5%

Adapted from Burri H et al. EHRA consensus statement on optimal implantation technique for conventional pacemaker and implantable cardioverter defibrillators [25]. These data reflect both pacemaker and defibrillator implantation. Cardiac resynchronization therapy was under-represented. Incidence ranges capture both single- and dual-chamber devices.

**Table 3:** Available Device Algorithms Designed to Optimize CRT Performance.

Algorithm	Manufacturer	Optimization	Mode	Programmable?	Dynamic?	Safety Endpoint Met?	Trial Efficacy Endpoint Met? *
AdaptivCRT [63]	Medtronic	AV, VV	IEGM	No	Yes (1/min)	Yes	Non-inferior
QuickOpt [64]	Abbott	AV, VV	IEGM	Yes	No	Yes	Non-inferior
SmartDelay [65]	Boston	AV	IEGM	No	No	Yes	Equivalent
SonR [66]	Sorin	AV, VV	Hemodynamic sensor	No	Yes (1/week)	Yes	Non-inferior
SyncAV [67]	Abbott	AV	IEGM	Yes	Yes (1/256 beats)	NR	NR
AutoAdapt ^†^	Biotronik	AV, VV	IEGM	NR	Yes (1/min)	NR	NR

IEGM = intracardiac electrograms; NR = Not reported; * Comparison versus conventional BiV pacing without device algorithms programmed on or versus echo-guided CRT optimization; ^†^ NCT04774523.

**Table 5:** Pivotal Trials of Leadless Pacing Devices.

Trial	Year	Device	N	Mean Age	% Female	Follow-up	Primary Outcome	Implant Success Rate, n/N (%)	Complication Rate	**Other**
LEADLESS [121]	2014	NanoStim	33	76.5 ± 8.4	33	3 m	31/33 (94%) *	32/33 (97%)	3% (1 perforation requiring surgery; died of stroke)	5 patients require > 1 LP during procedure
LEADLESS II [122]	2015	NanoStim	526	75.8 ± 12.1	38.2	6 m	270/300 (90.0%) ^†^	504/526 (95.8%)	6.7% (22 events in 20 patients)	1.7% dislodgements 1.3% tamponade 1.3% elevated thresholds
Micra IDE [123]	2016	Micra	725	75.9 ± 10.9	41.2	6 m	292/297 (98.3%) ^‡^	719/725 (99.2%)	4.0% (28 events in 25 patients)	1.6% perforation/effusion
LEADLESS II-Phase 2 [124]	2022	Aveir	200	75.6 ± 11.3	37.5	6 w	188/196 (95.9%) ^¶^	196/200 (98%)	4.0% (9 events in 8 patients)	1.5% tamponade

* Freedom from complications at 90 days; ^†^ Composite of acceptable pacing threshold (≤2 V at 0.4 ms) and acceptable sensing amplitude (R wave ≥5 mV) through 6 months; ^‡^ Percentage of patients with low and stable pacing capture thresholds (≤2 V at 0.24 ms and an increase of ≤1.5 V from time of implant) at 6 months; ^¶^ Composite of acceptable pacing thresholds (≤2 V at 0.4 ms) and R wave amplitudes (≥5 mV or an equal or greater value at implantation) through 6 weeks.

## Data Availability

Not applicable.
